# The bidirectional temporal relationship between headache and affective disorders: longitudinal data from the HUNT studies

**DOI:** 10.1186/s10194-022-01388-x

**Published:** 2022-01-21

**Authors:** Samita Giri, Erling Andreas Tronvik, Knut Hagen

**Affiliations:** 1 Norwegian Advisory Unit on Headaches, St. Olavs University Hospital, Trondheim, Norway; 2 Department of Neuromedicine and Movement science, Norwegian University of Science and Technology, Trondheim, Norway; 3 Clinical Research Unit Central Norway, St. Olavs University Hospital, Trondheim, Norway; 4 Department of Neuromedicine and Movement science, Faculty of Medicine, Norwegian University of Science and Technology, 7489 Trondheim, Norway

**Keywords:** Migraine, Epidemiology, General population, Follow-up

## Abstract

**Background:**

Few prospective population-based studies have evaluated the bidirectional relationship between headache and affective disorder. The aim of this large-scale population-based follow-up study was to investigate whether tension-type headache (TTH) and migraine had increased risk of developing anxiety and depression after 11 years, and vice-versa.

**Methods:**

Data from the Trøndelag Health Study (HUNT) conducted in 2006-2008 (baseline) and 2017-2019 (follow-up) were used to evaluate the bidirectional relationship between migraine and TTH and anxiety and depression measured by Hospital Anxiety and depression Scale (HADS). The population at risk at baseline consisted of respectively 18,380 persons with HADS score ≤ 7 and 13,893 without headache, and the prospective data was analyzed by Poisson regression.

**Results:**

In the multi-adjusted model, individuals with HADS anxiety (HADS-A) and depression scores (HADS-D) of ≥8 at baseline nearly doubled the risk of migraine (Risk rations (RR) between 1.8 and 2.2) at follow-up whereas a 40% increased risk (RR 1.4) was found for TTH. Vice versa, the risk of having HADS-A and HADS-D scores of ≥8 at follow-up were increased for TTH (RR 1.3) and migraine (RR 1.3-1.6) at baseline. Migraine with aura was associated with 81% (RR 1.81, 95% 1.52-2.14) increased risk of HADS-A score of ≥8.

**Conclusions:**

In this large-scale population-based follow-up study we found a bidirectional relationship between anxiety and depression and migraine and TTH. For anxiety, this bidirectional association was slightly more evident for migraine than TTH.

## Introduction

Migraine, depression, and anxiety are all ranked as top ten causes of years lived with disability, affecting the health and economy on individuals, and with high economic burden to most countries in Europe [1–3]. The association between affective disorders and migraine is well established in cross-sectional studies e.g. [4–8], whereas few longitudinal studies have evaluated the temporal bidirectional relationship between primary headaches and anxiety and depression, and with inconsistent results [9–14]. Methodological differences between studies e.g. regarding sample size and study population may partly explain inconsistent findings [13].

Among the few large-scale longitudinal cohort studies evaluating the bidirectional relationship, a Canadian study of 15,254 individuals reported that persons with migraine were 60% more likely to develop depression, and vice versa, that individuals with depression were 40% more likely to develop migraine [13]. On the other hand, a prospective study from the United States who did not find any association between depression and migraine, may have been underpowered to detect this relationship [11]. Little is known, however, about the relationship between migraine and anxiety, and between tension-type headache (TTH) and affective disorders.

The association between migraine and psychiatric comorbidities are complex, and influence the clinical course, treatment response and clinical outcomes [15, 16]. The complexity is highlighted in a recent review regrding the impact of traumatic life events and post-traumatic stress disorder on migraine patients [16]. Coexisting migraine and post-traumatic stress disorder affect the quality of life and most likely the course of post-traumatic headache [17].

The objective of this prospective large-scale population-based 11-year follow-up study was to evaluate whether anxiety and depression measured by Hospital Anxiety and Depression Scale are associated with increased risk of questionnaire-based diagnoses of migraine and TTH, and whether these primary headache disorders are associated with increased risk of anxiety and depression. Based on the previous studies, we hypothesized the existence of a bidirectional relationship between anxiety and depression and primary headache disorders, and that this relationship will be stronger for depression and migraine, respectively, than for anxiety and TTH.

## Methods

### Study design

This is a population-based historical prospective cohort study evaluating the bidirectional relationship between common primary headache disorders and affective disorders.

### The HUNT surveys

The present study used baseline data from the third Trøndelag Health study (HUNT3) performed 2006-2008 and follow-up data from the HUNT4 study performed 2017- 2019 [18–20]. In both surveys the entire population of the Nord-Trøndelag County aged ≥ 20 years was invited to answer many health-related items in two different questionnaires (Q1 and Q2), including Q2 questions about headache and anxiety and depression, and to participate in a clinical examination, including measurement of weight and height.

### Hospital anxiety and depression scale

HUNT3 and HUNT4 included the self-administered questionnaire Hospital Anxiety and Depression Scale (HADS), consisting of seven questions each about anxiety (HADS-A) and depression (HADS-D). Each question was scored on a scale of 0–3, yielding two subscales with a range of 0–21, with higher scores indicating higher levels of distress. The seven items of HADS-D are relevant for diagnostic criteria of depression in the fifth version of the Diagnostic and Statistical Manual of Mental Disorders (DSM-5) and ICD-11 (World Health organization, 2019), and most of the items of HADS-A are relevant for criteria of generalized anxiety disorder (GAD) for both diagnostic manuals. A study based on HUNT2 (*n*=52,265) found that HADS was a suitable instrument for assessment in the general population [21]. A review of previous studies has concluded that the cutoff score of 8 give an optimal balance between sensitivity and specificity, both of which are around 0.80 on both subscales [22], whereas the ≥11 cut-off value increases the specificity. In the present study, individuals with HADS-A and HADS-D score between 0 and 7 constituted the reference group (0-14 for total HADS score). These were compared with HADS-A and HADS-D score of ≥8 (≥15 for total HADS score). In addition, as recommended by Zigmond and Snaith [23], we also divided the participants with elevated HADS score into two groups: 8-10 indicating possible cases of anxiety or depression, and ≥11 as probable cases (15-21 and ≥22 for total HADS score). The total HADS score are reflecting those with combined depression and anxiety. In the data file distributed by the HUNT research Centre, the total HADS score was extrapolated for those with at five or six completed items on both the HADS-D and the HADS-A, and the sum of completed items were multiplied with 7/5 or 7/6, respectively.

### Headache diagnoses

HUNT3 and HUNT4 had 14 identical headache questions [18]. The initial screening question was “Have you suffered from headache during the last 12 months?”, and those who responded “yes” answered 13 additional questions. Individuals who answered “no” in HUNT3 were included in the population at risk of headache in HUNT4. A slight modification of the criteria of the International Classification of Headache Disorders, third edition (ICHD 3) [24] was used for diagnosing migraine, probable migraine, and tension-type headache (TTH). The diagnoses were mutually exclusive. As to the modifications of the criteria, individuals would fulfill the migraine criteria even if the attack lasted less than 4 hours, because they were not asked for untreated attacks in the question ”How long does the headache usually last?”

We have previously reported the validity of the questionnaire-based headache diagnoses in HUNT3 [25] and HUNT4 [26]. Merged data of two studies based on HUNT3 (*n*=293) and HUNT4 (*n*=232) gave the following results; for definitive migraine, the sensitivity was 63% and specificity 92% (kappa values 0.57, 95% CI 0.44-0.70), for migraine with aura, the sensitivity was 39% and specificity 95% (kappa values 0.34, 95% CI 0.30-0.38), and or TTH ≥1 days/month, the sensitivity was 97% and specificity was 71% (kappa value 0.38, 95% CI 0.31-0.45) [18, 25, 26].

### Study population

**Fig. 1:**
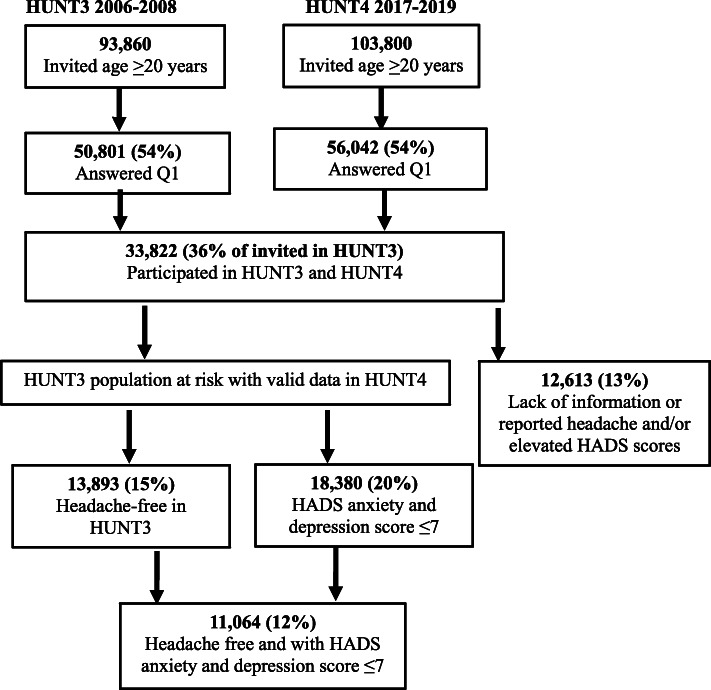
The flow of participants in HUNT3 and HUNT4.

The numbers of invited individuals and participants in HUNT3 and HUNT4 are presented in the flow diagram (Fig. 1). Out of 50,801 HUNT3 participants (54% of invited), 33,822 (36%) also participated in HUNT4. In HUNT4, 56,042 (54%) aged ≥20 years participated out of 103,800 invited (Fig. 1). Among those who were invited, 41,460 (40%) answered the headache questions and 40,996 (39%) had HADS scores.

Participants for the present study were selected based on the following two inclusion criteria: (1) Participated in HUNT3 and HUNT4 and had answered questions related to headache, anxiety, and depression in both surveys (*n*=21,209); (2) Headache free at baseline in HUNT3 (population at risk of developing headache in HUNT4, *n*=13,893, 15%) or had HADS score ≤7 in HUNT3 (population at risk for elevated HADS score in HUNT4, *n*=18,380, 20%) (Fig. 1). HUNT-participants who lacked information on headache and/or HADS scores (*n*=12,613, 12%) were excluded from the present study (Fig. 1).

### Confounders

The selection of potential confounders was based on a review of previous population-based studies [27–34]. In the HUNT studies we have previously demonstrated that primary headache disorders and affective disorders both have a strong relationship with age and sex [27, 28], and independent associated with socioeconomic status [29, 30], smoking [31–33] and other lifestyle factors [34]. Thus, in the present study we evaluated the following potential confounders: Age (10-years categories), sex, duration of education (≤9, 10-12, and ≥13 years), body mass index (BMI) (<25, 25.0-29.9, and ≥30 kg/m^2^), and smoking (current, previous, and never).

### Ethics

This study was approved by the Regional Committee for Medical and Health Research Ethics, the Faculty of medicine, mailbox 8905, 7491 Trondheim. The approval number was #2018/2422/Rek Midt. The participants have given written informed consent.

### Statistical analysis

Descriptive analyses of the study population are presented through numbers and percentages for categorical variables and means and standard deviations for continuous variables.

A modified Poisson regression with a robust error variance was used to estimate the following: In the headache free group at baseline in HUN3 (defined as population at risk of headache at follow-up), the association between HADS score at baseline and risk ratios (RRs) of definitive and probable migraine and TTH were evaluated. Furthermore, in the group with HADS score of ≤7 (defined as the population at risk of having elevated HADS score at follow-up), the association between headache diagnoses at baseline and risk ratios (RRs) of elevated HADS score was investigated. Precision of RRs were assessed by 95% confidence intervals (CIs). We present results for two different statistical models separated by the number of categorical potential confounders included. Model 1 included age, sex, and education level, in model 2 smoking status and BMI were added. Subjects with missing data on smoking status and BMI (Table 1) were included in the analysis to reduce the impact of possible bias. Analyses were performed with the IBM SPSS version 26 (SPSS, Chicago, Illinois, USA).

**Table 1:** Baseline characteristics of population at risk in HUNT3 with valid data in HUNT4 defined as respectively without headache and with anxiety and depression HADS^1^ score of ≤7

	Population at risk in HUNT3	
Characteristics	Without headache	HADS score *≤*7
Number of participants with valid data in HUNT4	13,893	18,380
Women (%) (missing=0)	52.6	57.6
Mean age, years (SD) (missing=0)	54.6 (13.1)	52.3 (13)
≥13 years of education (%) (missing=0)	36.1	38.8
Current smoking, n (%) (missing=308/363)	12.6	12.9
Mean BMI^2^, kg/m^2^ (SD) (missing=16/28)	27.1 (4.1)	27.1 (4.2)
Mean HADS-A^3^ (SD) (missing=303/0)	3.5 (3.0)	2.9 (2.1)
Mean HADS-D^4^ (SD) (missing=247/0)	3.0 (2.7)	2.4 (2.0)
Mean total HADS^1^ score (missing=0)	6.5 (4.9)	5.3 (3.3)
Alcohol abstainers last year (%) (missing=243/287)	6.6	6.1
Self-reported hypertension, n (%) (missing=0)	21.2	18.4
Self-reported stroke (%) (missing=0)	1.8	1.6
Self-reported diabetes mellitus, n (%) (missing=0)	4.2	3.5
Headache (%) (missing=0)	0	35.7

^1^HADS=Hospital Anxiety and Depression Scale; ^2^BMI=body mass index; ^3^HADS-A: Anxiety score; ^4^HADS-D: Depression score

## Results

Baseline characteristics of the HUNT3 population at risk without headache (*n*=13,893) and with HADS anxiety and depression score ≤7 (*n*=18,380) are presented in Table 1. A total of 11,064 (80% of those without headache) also had HADS score ≤7 (Fig. 1). Mean BMI was identical in both population at risk groups. On the other hand, compared to the headache free group, individuals with HADS score ≤7 were younger (mean age 52.3 vs. 54.6 years) and more likely to be women (57.6% vs. 52.6%). Furthermore, those with HADS score ≤7 were less likely to report hypertension, stroke, and diabetes mellitus than those without headache (Table 1). Among women without headache (*n*=7,307) or with HADS anxiety and depression score ≤7 (*n*=10,587), 2% reported to be pregnant in HUNT3 and 86% reported having ≥1 child.

### The impact of anxiety and depression on the risk of headache in HUNT4

Among the 13,893 persons without headache in HUNT3, 273 (2.0%) developed definite migraine, 1438 (10.3%) TTH and 229 (1.7%) probable migraine. In the fully adjusted model, HADS-A score of ≥8 nearly doubled the risk of having definite migraine (RR 1.75, 95% 1.27-2.42) and probable migraine (RR 1.76, 95% 1.24-2.49) whereas a 44% increased risk was found for TTH (RR 1.44, 95% CI 1.22-1.70) (Table 2). For HADS-D score of ≥8 higher risk were found for definite migraine (RR 2.15, 95% CI 1.45-3.18) and TTH (RR 1.48, 95% 1.23-1.79), but not evident for probable migraine (RR 1.45, 95% 0.90-2.35). Individuals with combined anxiety and depression (total HADS score ≥22 at baseline) had more than four times increased risk of developing definite migraine (RR 4.54, 95% CI 2.39-8.60) and three times increased risk of developing probable migraine (RR 3.20, 95% CI 1.44-7.13), whereas the RR of TTH was 1.64 (95% CI (1.11-2.41) (Table 2).

**Table 2:** Headache free individuals in HUNT3 (*n*=13,893): Risk of migraine and tension-type headache in HUNT4 based on HADS^1^ score categories in HUNT3 evaluated by poisson regression with 95% confidence interval

	Number	Definite migraine (*n*=273)	Tension-type headache (*n*=1436)	Problable migraine (*n*=229)
		RR (95% CI)	RR (95% CI)	RR (95% CI)
**Model 1** (Adjusted for age, gender and education level)				
HADS-A^2^ ≤ 7	12,276	1.00	1.00	1.00
8-10	936	1.82 (1.27-2.61)	1.45 (1.23-1.71)	1.90 (1.28-2.80)
≥11	378	1.77 (0.98-3.19)	1.41 (1.11-1.80)	1.65 (0.88-3.09)
HADS-D^3^ ≤ 7	12,677	1.00	1.00	1.00
8-10	777	2.20 (1.43-3.38)	1.49 (1.24-1.80)	1.45 (0.85-2.48)
≥11	192	2.28 (1.01-5.14)	1.11 (0.72-1.69)	1.80 (0.68-4.74)
Total^4^ ≤ 14	12,877	1.00	1.00	1.00
15-21	871	1.81 (1.19-2.74)	1.57 (1.33-1.86)	1.42 (0.87-2.30)
≥22	145	4.89 (2.59-9.24)	1.70 (1.17-2.48)	3.34 (1.51-7.38)
**Model 2** (Adjusted for age, gender, education level, smoking status and body mass index)				
HADS-A^2^ ≤ 7	12,276	1.00	1.00	1.00
≥8	1314	1.75 (1.27-2.42)	1.42 (1.23-1.64)	1.76 (1.24-2.49)
8-10	936	1.77 (1.24-2.53)	1.44 (1.22-1.70)	1.83 (1.24-2.72)
≥11	378	1.71 (0.95-3.09)	1.38 (1.08-1.77)	1.59 (0.85-2.98)
HADS-D^3^ ≤ 7	12,677	1.00	1.00	1.00
≥8	969	2.15 (1.45-3.18)	1.41 (1.18-1.67)	1.45 (0.90-2.35)
8-10	777	2.13 (1.38-3.29)	1.48 (1.23-1.79)	1.39 (0.81-2.38)
≥11	192	2.21 (0.99-4.92)	1.11 (0.72-1.70)	1.79 (0.67-4.76)
Total^4^ ≤ 14	12,877	1.00	1.00	1.00
≥15	1016	2.10 (1.46-3.04)	1.57 (1.34-1.84)	1.58 (1.03-2.43)
15-21	871	1.74 (1.14-2.66)	1.56 (1.32-1.85)	1.35 (0.83-2.21)
≥22	145	4.54 (2.39-8.60)	1.64 (1.11-2.41)	3.20 (1.44-7.13)

^1^Hospital Anxiety and Depression Scale score; ^2^HADS-A: Anxiety score; ^3^HADS-D: Depression score; ^4^Total HADS score

### The impact of headache on the risk of elevated anxiety and depression score in HUNT4

Among the 18,380 individuals with HADS-A and HADS-D score ≤7 at baseline in HUNT3, 1228 (6.7%) had HADS-A score between 8 and 10 and 349 (1.9%) had HADS-A score ≥11, whereas the corresponding figures for HADS-D was 751 (4.1%) and 182 (1.0%) (Table 3). 822 (4.6%) out of 18,380 individuals reported prescription of medication for anxiety or depression in HUNT4. Table 3 shows the impact of headache types in HUNT3 on the risk of two separate elevated HADS score groups in HUNT4, whereas Table 4 present merged data. In the fully adjusted analyses in Table 4 (model 2), the risk of HADS-A score of ≥8 was increased for individuals with definite migraine (RR 1.59, 95% CI 1.38-1.82), migraine without aura (RR 1.39, 95% CI 1.15-1.67), migraine with aura (RR 1.81, 95% CI 1.52-2.14), probable migraine (RR 1.30, 95% CI 1.09-1.56) and TTH (RR 1.29, 95% CI 1.14-1.47) (Table 4). The risk of HADS-D score of ≥8 was increased for definite migraine (RR 1.26, 95% CI 1.02-1.56) and TTH (RR 1.30, 95% CI 1.10-1.54). Finally, the risk of having the combination of anxiety and depression (total score of ≥15) was increased for definite migraine (RR 1.42, 95% CI 1.17-1.71), migraine with aura (RR 1.67, 95% CI 1.32-2.10), probable migraine (RR 1.38, 95% CI 1.11-1.72) and TTH (RR 1.18, 95% CI 1.00-1.39).

**Table 3:** HADS^1^ anxiety and depression score ≤ 7 in HUNT3 (*n*=18,380): Risk of elevated HADS^1^ score in HUNT4 based on headache diagnoses in HUNT3 evaluated by poisson regression with 95% confidence interval

	Number	HADS anxiety score		HADS depression score		Total HADS score	
		8-10 (n=1228)	≥ 11 (n=349)	8-10 (n=751)	≥ 11 (n=182)	15-21 (n=862)	≥ 22 (n=131)
		RR (95% CI)	RR (95% CI)	RR (95% CI)	RR (95% CI)	RR (95% CI)	RR (95% CI)
**Model 1:** Adjusted for age, gender and education level							
Headache free	11,819	1.00	1.00	1.00	1.00	1.00	1.00
TTH^2^	3063	1.34 (1.10-1.55)	1.19 (0.90-1.57)	1.40 (1.16-1.69)	0.93 (0.60-1.43)	1.21 (1.01-1.44)	1.04 (0.64-1.68)
Definite migraine	1962	1.71 (1.46-2.00)	1.33 (0.97-1.81)	1.27 (1.00-1.63)	1.36 (0.87-2.12)	1.52 (1.24-1.85)	1.10 (0.63-1.91)
Without aura	1038	1.61 (1.31-1.96)	0.72 (0.42-1.21)	1.35 (0.98-1.85)	0.86 (0.42-1.77)	1.34 (1.02-1.76)	0.39 (0.12-1.29)
With aura	924	1.83 (1.50-2.29)	2.02 (1.42-2.88)	1.19 (0.84-1.70)	1.91 (1.13-3.22)	1.71 (1.31-2.21)	1.90 (1.04-3.35)
Probable migraine	1252	1.32 (1.07-1.62)	1.33 (0.91-1.94)	1.19 (0.90-1.59)	1.24 (0.72-2.18)	1.45 (1.14-1.85)	1.13 (0.59-2.21)
**Model 2:** Adjusted for age, gender, education level, smoking status and body mass index							
Headache free	11,819	1.00	1.00	1.00	1.00	1.00	1.00
TTH^2^	3063	1.34 (1.16-1.57)	1.18 (0.89-1.56)	1.40 (1.16-1.68)	0.92 (0.59-1.42)	1.21 (1.01-1.44)	1.03 (0.64-1.67)
Definite migraine	1962	1.69 (1.45-1.98)	1.29 (0.94-1.77)	1.25 (0.98-1.61)	1.29 (0.82-2.02)	1.49 (1.22-1.82)	1.00 (0.57-1.74)
Without aura	1038	1.61 (1.31-1.96)	0.72 (0.42-1.21)	1.35 (0.98-1.85)	0.86 (0.42-1.77)	1.34 (1.02-1.76)	0.39 (0.12-1.29)
With aura	924	1.83 (1.50-2.29)	2.02 (1.42-2.88)	1.19 (0.84-1.70)	1.91 (1.13-3.22)	1.71 (1.31-2.21)	1.90 (1.04-3.35)
Probable migraine	1252	1.30 (1.06-1.60)	1.31 (0.89-1.92)	1.19 (0.89-1.58)	1.24 (0.71-2.17)	1.44 (1.13-1.82)	1.11 (0.57-2.16)

^1^Hospital Anxiety and Depression Scale; ^2^Tension-type headache

**Table 4:** HADS^1^ anxiety and depression score ≤ 7 in HUNT3 (*n*=18,380): Risk of elevated HADS^1^ score in HUNT4 based on headache diagnoses in HUNT3 evaluated by poisson regression with 95% confidence interval

	Number	HADS-A ≥ 8 (*n*=1577)	HADS-D ≥ 8 (*n*=933)	Total HADS≥ 15 (*n*=993)
		RR (95% CI)	RR (95% CI)	RR (95% CI)
**Model 1**				
Headache free	11,819	1.00	1.00	1.00
TTH^2^	3063	1.30 (1.14-1.47)	1.30 (1.10-1.54)	1.18 (1.00-1.40)
Definite migraine	1962	1.60 (1.36-1.83)	1.29 (1.04-1.60)	1.45 (1.20-1.75)
*Without aura*	*1038*	*1.39 (1.15-1.67)*	*1.24 (0.93-1.65)*	*1.20 (0.93-1.56)*
*With aura*	*924*	*1.83 (1.55-2.17)*	*1.36 (1.02-1.81)*	*1.73 (1.37-2.18)*
Probable migraine	1252	1.31 (1.10-1.56)	1.20 (0.93-1.54)	1.39 (1.12-1.74)
**Model 2**				
Headache free	11,819	1.00	1.00	1.00
TTH^2^	3063	1.29 (1.14-1.47)	1.30 (1.10-1.54)	1.18 (1.00-1.39)
Definite migraine	1962	1.59 (1.38-1.82)	1.26 (1.02-1.56)	1.42 (1.17-1.71)
*Without aura*	*1038*	*1.39 (1.15-1.67)*	*1.22 (0.91-1.62)*	*1.18 (0.99-1.39)*
*With aura*	*924*	*1.81 (1.52-2.14)*	*1.31 (0.99-1.62)*	*1.67 (1.32-2.10)*
Probable migraine	1252	1.30 (1.09-1.56)	1.19 (0.92-1.53)	1.38 (1.11-1.72)

^1^Hospital Anxiety and Depression Scale score; ^2^Tension-type headache

## Discussion

In this large-scale population-based study a bidirectional relationship between anxiety and depression with migraine and TTH was found. We identified slightly higher risk ratio for definite migraine than for TTH for anxiety.

### Comparison with previous studies

In the present study we found approximately 30% increased risk of depression among patients with migraine or TTH, and that depression at baseline more than doubled risk of migraine, whereas a 40% increased risk of TTH was found. Accordingly, a questionnaire-based 14-year follow-up study of 36,016 women without depression at baseline, reported that the risk of depression was 1.4 (1.3-1.6) for non-migrainous headache, 1.5 (1.4-1.8) for migraine with aura, 1.4 (1.3-1.6) for migraine without aura, and 1.6 (1.4-1.8) for past history of migraine [14]. Most of the previous population-based follow-up studies from Canada and US have focused mainly on the temporal relationship between migraine and major depression. For example, a 12-year population-based follow-up of approximately 14,000 individuals aged >12 years from Canada demonstrated a bidirectional relationship between migraine and major depressive episodes [13]. Thus, migraine gave a 60% increased risk (RR 1.6, 95% CI 1.3-1.9) of major depression, whereas major depression gave 40% increased risk (RR 1.4, 95% CI 1.0-1.9) migraine [13]. A separate publication based on this Canadian population focused on 8-year follow-data of 9,288 participants aged 18-64 years [35]. In sex and age-adjusted analyses they showed that depression was predictive of migraine (HR 1.62, 95% CI 1.03 - 2.53) and migraine was predictive of depression (HR 1.55, 95% CI 1.15 - 2.08) [34]. Interestingly, however, supplementary adjustment for major life stressors e.g. childhood trauma and/or work stress decreased the association substantially [35]. Another two-year follow-up study of adults aged 25-55 years in the US showed a bidirectional association between major depression and migraine [36]. Major depression at baseline predicted the first-onset migraine (OR 3.4, 95% CI 1.4-8.7), and vice versa, migraine at baseline predicted the first-onset major depression (OR 5.8, 95% CI 2.7-12.3) [36].

Some previous cross-sectional studies have diagnosed depression using HADS score. Depression defined as HADS-D score of ≥ 11 was more than two times more likely among individuals with migraine than non-migrainous headache based on cross-sectional data from HUNT2 [5]. Similarly, merged data of six previous EU-based cross-sectional studies reported that migraine was twice as common (OR 2.1, 95% CI 1.3–3.4) among individual with depression measured by HADS [37]. A recent meta-analysis based on data from cohort studies reported that migraineurs were almost twice (OR 1.81, 95% CI 1.20-2.72) as likely to develop depression as those without migraine [38].

In the present study, migraine with aura was associated with higher RR (1.7) of co-existing depression and anxiety than RR (1.3) of depression alone. A previous cross-sectional study showed that individuals with migraine with aura were more likely to be depressed compared to migraine without aura in women but not in men [8]. In the Michigan study, the risk for depression was almost twice as likely among migraine with aura compared to migraine without aura (ORs 4.0 vs. 2.2) [39].

A bidirectional association between depression and TTH was also found in the present study. To our knowledge, data on such a relationship has not been provided for TTH in previous longitudinal studies. In cross-sectional population-based studies, divergent results have been reported for TTH. A recent population-based study performed in Korea reported that patients with TTH were almost two times more likely to have depression (4.2% vs. 1.8%) than those without [40]. In contrast, a Nepalese population-based study found no association between depression and TTH [41]. Similarly, no association was found between depression measured by HADS and TTH in analyses using the merged data of six previous cross-sectional EU studies [37].

In the present study, we found that anxiety increased the risk of migraine, probable migraine, and TTH, and vice versa, that all these headache types increased the risk of anxiety.

Very few long-term prospective studies have evaluated the temporal bidirectional relationship between anxiety disorders and common primary headache disorders. Notably, prospective data of 591 young adults from Switzerland showed that the combination of major depression and anxiety disorder doubled the risk of migraine, and that anxiety disorders generally preceded the occurrence of migraine [9]. Furthermore, in a 1.2-year follow-up study of 1,007 adults aged 21-30 years, migraine at baseline increase the odds ratio of panic disorders with 12.8 [10]. Several cross-sectional population-based studies have reported evidence for positive associations between migraine and anxiety [5, 41–44]. In studies from Norway, the US and Canada, anxiety disorders were respectively 3.2, 3.1 and 2.5 times higher among migraineurs compared to headache-free individuals [5, 42, 43]. A recent systematic review evaluated the comorbidity of anxiety and migraine in the cohort studies, and estimated RR with an average of 1.63 [45].

In the present study, higher risk of anxiety was found for migraine with aura than for migraine without aura (1.8 vs. 1. 39). In accordance with the present study, depression with comorbid anxiety was more likely among women with migraine with aura than among those without aura in a cross-sectional study based on HUNT2, whereas an association between anxiety alone and migraine with aura was not found [8]. In the Michigan study, the association between anxiety and migraine was slightly higher for migraine with aura than for migraine without aura (ORs 3.1 vs. 2.3) [39].

Very few population-based cross-sectional studies have evaluated the impact of anxiety on TTH. A Korean population-based study reported that the prevalence of anxiety was almost twice as high (9.5% vs. 5.3%) among patients with TTH compared to headache-free controls [40]. Furthermore, TTH was associated with anxiety measured by HADS score both for males (ORs 2.5, 95% CI 1.7–3.7) and females (OR 1.5, 95% CI 1.1–2.1) based on data from six EU studies [37]. In the present study, a bidirectional relationship between co-existence of anxiety and depression (defined as total HADS score of ≥15 or ≥22) was more evident for migraine than for TTH. For example, total HADS score ≥22 at baseline was associated with more than four times increased risk of migraine at follow-up. In accordance, comorbidity of anxiety and depression was more strongly associated with increased risk of migraine than pure anxiety or depression in the Zurich study [44]. Furthermore, migraine was associated with increased OR of major depression combined with panic disorders 25.2 (95% CI 2.5-251) in the 1.2-year US follow-up study, whereas OR of 2.5 (95% CI 1.0-6.5) was found for major depression alone [10].

### Interpretation

The bidirectional relationship between affective disorders and migraine and TTH could be the results of shared pathophysiological mechanisms related to neurotransmitters, genetic basis and/or environmental factors. The follow-up studies from Canada demonstrated that life stressors such as childhood trauma may be an important common underlying factor, supporting the environmental aspect [13, 35]. Most previous studies focusing on the relationship between migraine and depression, suggest a common dysfunction related to serotonin, dopaminergic and GABAergic systems [46, 47]. Furthermore, twin and family studies have indicated that the bilateral relationship between migraine and depression at least in part could be explained by genetic factors [46, 47, 49]. However, it is still unclear how specific genetic variants are associated with both the primary headaches and affective disorders. In the present study, we identified a bidirectional relationship between anxiety and depression with migraine and TTH. Thus, in the diagnostic headache interview, clinicians should take into account the existence of psychiatric comorbidities and consider to include questions regarding traumatic life events. Potential beneficial or synergistic effects as well as treatment complications should be considered when choosing treatment [16]. Vice versa, headache questions should be included when assessing patients admitted for psychiatric evaluation, and comorbid headache should be taken into account during treatment.

### Strengths and limitations of the study

The major strengths of this study are the prospective design, an 11-year follow-up period, and the large cohort from the adult population of an entire population of Trøndelag County. The present study design is more likely to identify causal relationships than cross-sectional studies. Furthermore, in the longitudinal analysis of risk factors at baseline, we have adjusted for the same confounding factors in all analyses, making the estimated RRs comparable. Finally, the study included use of validated diagnoses of headache [25, 26] and use of well-established cutoff score of HADS [21–23]. However, since no previous longitudinal studies have evaluated depression or anxiety using HADS score, direct comparison with these prospective studies should be done with caution.

Several study limitations should also be addressed. Firstly, generalization of the results to the entire population must be made with caution, since the participation rate was 54% and 58% respectively in the two surveys, and only 36% of the invited population in HUNT3 participated in HUNT4. On the other hand, a bias in relative risk estimates will only arise if participation rates differ regarding headache status or affective disorders. The fact that neither headache nor anxiety and depression were primary objectives of these surveys makes selective participation and loss follow-up unlikely. Secondly, the diagnostic accuracy of questionnaire-based diagnoses of headache and affective disorders was not optimal, and the possibility of misclassification cannot be ruled out. However, most likely, such misclassification goes in both directions, decreasing the difference in RR between the diagnostic subgroups. The gold standard for the diagnosis of common primary headache disorders and affective disorders is the face-to-face interview by experts, but such strategy is not possible in large-scale population-based studies. Thirdly, even prospective studies are more likely to identify causal relationships than cross-sectional studies, the present study lacked information on the time of onset of both depression/anxiety and migraine/TTH, which would allow identification of causal relationship between both disorders. Finally, the number of individuals with HADS score ≥11 were relatively low. However, the associations studied were sufficiently powered to detect significant differences using the cut-off score ≥8 and for the 8-10 HADS score groups.

## Conclusions

In this large-scale population-based follow-up study, we found a bidirectional relationship between anxiety and depression and migraine and TTH. This result may indicate shared underlying pathophysiological mechanisms related to neurotransmitters, genetic basis and/or environmental factors, and that effective management of affective disorders may hold the potential to reduce the incidence of migraine and TTH, and vice versa.

## Data Availability

Part of the dataset supporting the conclusions of this article is available on request to the corresponding author. Some of the data are the property of HUNT research Centre and can only be accessed through direct contact with. the research Centre.

## Abbreviations

BMI: Body mass index
CI: Confidence interval
DSM-5: Fifth version of the Diagnostic and Statistical Manual of Mental Disorders
GAD: Generalized anxiety disorder
HADS: Hospital Anxiety and depression Scale
HADS-A: Anxiety
HADS-D: Depression
HUNT: The Trøndelag Health Study
HUNT2: The second Trøndelag Health Study
HUNT3: The third Trøndelag Health Study
HUNT4: the fourth Trøndelag Health Study
RR: Risk ratio
TTH: Tension-type headache
